# Enhanced Macrophage Tribbles-1 Expression in Murine Experimental Atherosclerosis

**DOI:** 10.3390/biology1010043

**Published:** 2012-04-10

**Authors:** Hye Youn Sung, Sheila E. Francis, Nadine D. Arnold, Karen Holland, Vanessa Ernst, Adrienn Angyal, Endre Kiss-Toth

**Affiliations:** Department of Cardiovascular Science, University of Sheffield, Sheffield, S10 2RX, UK; E-Mails: dowellyoun@yahoo.co.kr (H.Y.S.); s.francis@sheffield.ac.uk (S.E.F.); n.d.arnold@sheffield.ac.uk (N.D.A.); k.holland@sheffield.ac.uk (K.H.); vanessa.ernst@fh-krems.eu (V.E.); a.angyal@sheffield.ac.uk (A.A.)

**Keywords:** Tribbles, atherosclerosis, macrophages, mouse

## Abstract

Development of the atherosclerotic plaque involves a complex interplay between a number of cell types and an extensive inter-cellular communication via cell bound as well as soluble mediators. The family of tribbles proteins has recently been identified as novel controllers of pro-inflammatory signal transduction. The objective of this study was to address the expression pattern of all three tribbles proteins in atherosclerotic plaques from a mouse model of atherosclerosis. Each tribbles were expressed in vascular smooth muscle cells, endothelial cells as well as in resident macrophages of mouse atherosclerotic plaques. The role of IL-1 mediated inflammatory events in controlling tribbles expression was also addressed by inducing experimental atherosclerosis in ApoE^−/−^IL1R1^−/−^ (double knockout) mice. Immunohistochemical analysis of these mice showed a selective decrease in the percentage of trb-1 expressing macrophages, compared to the ApoE^−/−^ cohort (14.7% ± 1.55 *vs.* 26.3% ± 1.19). The biological significance of this finding was verified *in vitro* where overexpression of trb-1 in macrophages led to a significant attenuation (~70%) of IL-6 production as well as a suppressed IL-12 expression induced by a proinflammatory stimulus. In this *in vitro* setting, expression of truncated trb-1 mutants suggests that the kinase domain of this protein is sufficient to exert this inhibitory action.

## 1. Introduction

Atherosclerosis arises as a result of chronic inflammation of the artery wall, which occurs in response to a wide range of different stimuli, including fatty diets. The generation of atherosclerotic plaques involves a complex interplay between various vascular cell types and cytokine signalling networks. Although the molecular events that initiate the development of atherosclerotic lesions are still somewhat unclear, a large body of evidence supports a role for low-density lipoproteins (LDL), free radicals, infections [1], immune responses and biomechanical stress [2]. Many of the signals generated are processed via the mitogen activated protein kinase (MAPK) cascades, which are well known for acting as molecular controllers of cellular behaviour and their activation can lead to a diverse set of cellular functions. In particular, JNK kinases [3] and p38 kinases [4] have been shown to be highly active in smooth muscle cells (VSMC) in atherosclerotic lesions. In this context, MAPK activation is a substantial driver of plaque development as it promotes the production of pro-inflammatory cytokines and matrix metalloproteinases, induces the chemotactic migration of VSMC and monocytes as well as promoting VSMC proliferation. Therefore, intervention strategies with a clinical utility, which modulate plaque development via inhibiting MAPK pathways, are being sought continuously.

In recent years, a family of negative regulators of MAPK and PI3K cascades, called tribbles (trb) have emerged. These are evolutionally conserved, kinase-like proteins; their catalytic domain is missing some of the conserved residues thought to be indispensable for kinase activity [5]. They have been shown to interact with several transcription factors (reviewed in [5]) as well as MAPK-kinases (MKKs) [6] and Akt [7]. A controlling role for tribbles has been implicated in cell division [8,9] as well as in hormone induced cellular responses, including insulin [10] and pro-inflammatory cytokines [6].

Human genetic evidence suggests that trb-1 may be a risk factor in the development of hyperlipidemia and cardiovascular disease [11], whilst trb-3 has been linked to insulin resistance and type 2 diabetes [12]. In addition to these studies, our group has recently characterised the potential functional role of tribbles in key vascular cell types, vascular smooth muscle cells [13] and monocytes [14]. These experiments demonstrated that trb-1 controls the proliferation rate and chemotaxis of VSMC via interaction with the MAPK kinase, MKK4. Trb-2 is a key controller of inflammatory activation of monocytes and its’ expression is controlled by modified LDL. We have shown that trb-1 is expressed primarily in the intimal region of human atherosclerotic arteries, whilst Deng and colleagues have recently reported that trb-2 may be overexpressed in macrophages resident in unstable regions of atherosclerotic plaques, based on correlative quantitative PCR data [15].

However, whilst an important regulatory function for tribbles in vascular cell inflammation is supported by a number of studies and some expression data is available for individual tribbles homologues, no systematic study investigating the expression of the tribbles protein family has been performed in atherosclerosis.

In the study presented here, we have characterised the expression profile of all three mammalian tribbles in the ApoE^−/−^ murine experimental model of atherosclerosis. We have also identified the potential regulation of tribbles expression by IL-1, a master cytokine involved in atherosclerotic plaque development, *in vivo*. Our data demonstrate that smooth muscle cells and plaque resident macrophages express all three tribbles proteins. Using an Apo E/IL-1R double deficient mouse [16], we show that trb-1 expression in the macrophages is largely IL-1 dependent. *In vitro* studies confirm this finding, using Raw 267.4 mouse macrophages and show that trb-1 expression is elevated in response to LPS, a proinflammatory stimulus that signals via a largely identical set of signalling pathways to IL-1, and that this protein controls LPS induced IL-6 production in these cells.

## 2. Results and Discussion

### 2.1. Trb 1 Expression in Mouse Atherosclerotic Lesions

We have previously reported a selective increase in trb-1 expression in human aortic vascular smooth muscle cells within human atherosclerotic lesions compared to non-diseased aorta [13]. However, there is very limited information published to date on the expression of other tribbles in diseased arteries at the protein level. Therefore, we have studied here the expression pattern of trb-1, -2 and -3 in mouse atherosclerosis by immunostaining lesions obtained from the aortic root of fat-fed animals. Mice were fed a Western-type diet for 8 weeks whereupon they develop significant and complex lesions throughout the arterial tree as previously described [16]. Aortic root lesions were immunostained for trb-1 positive cells. In some sections, lesions were also stained for smooth muscle cells and macrophages.

**Figure 1:**
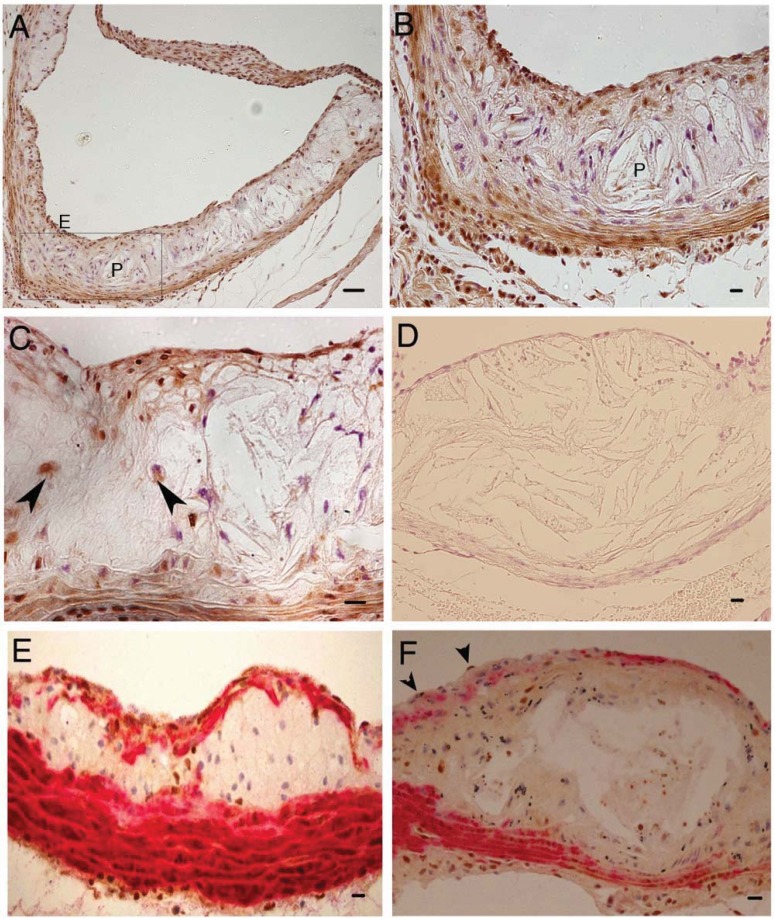
Tribbles 1 (trb-1) expression in experimental mouse atherosclerosis. **A** Trb1 expression (brown) in the aortic sinus P (plaque), E (endothelium); **B** High power of boxed inset from A, P (plaque); **C** Trb-1 expression in endothelium, some macrophages (arrows); **D** negative control (secondary Ab only); **E** dual staining for trb-1 (brown) and Smooth Muscle Cell Actin (red); **F** as E with some trb staining in endothelium (arrows). Note: some cells express trb-1 but not SMA and are probably macrophages.

Figure 1 shows the pattern of trb-1 expression in the aortic root of ApoE^−/−^ mice fed an atherogenic diet for 8 weeks. 

The majority of trb-1 protein is detected as expected in the medial region (Figure 1A and B) and in the fibrous cap (Figure 1A and C). When dual immunostaining is performed, the majority of trb-1 expression co-localises with smooth muscle actin (Figure 1E and F, trb-1 (brown)/actin (red)). In addition, some cells, particularly in the plaque areas express trb-1 but are not smooth muscle cells (Figure 1F).

**Figure 2:**
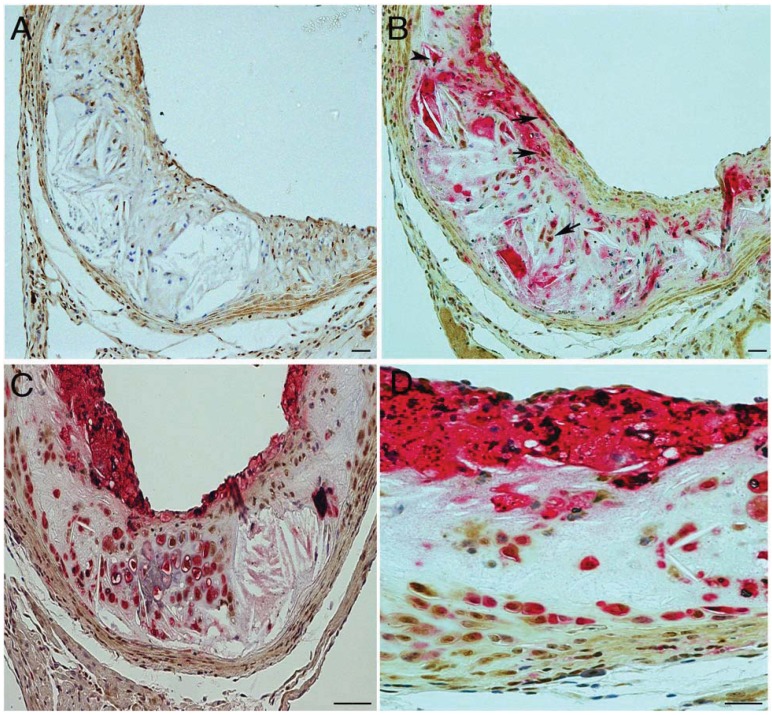
Tribbles 1 expression in macrophages in experimental mouse atherosclerosis. **A **Single immunostaining for trb-1 (brown) in a section from the aortic sinus, **B** Dual immunostaining of a serial section to A for trb1 (brown) and macrophages (Gal3, red), note areas of plaque stained red; trb-1 expressing macrophages are denoted by arrows. **C** and **D**, high power images of other representative plaques showing most macrophages stain positive for trb-1 in fat-fed ApoE deficient mice.

Numerous macrophages and some large plaques are also routinely detected in the aortic roots of fat-fed ApoE^−/−^ mice ([16] and Figure 2A). It is apparent that numerous trb-1 stained macrophages (macrophage (red), trb-1 (brown)) are present in these areas with a high plaque burden (Figure 2A shows trb1 staining alone, Figure 2B dual staining and 2C and 2D dual high power staining). The macrophage index—the number of macrophages/total number of cells × 100 in a 100 micron squared area of atherosclerotic plaque—for the vessel wall in these experiments was 51.3 ± 4.1%, n = 5. In addition, the percentage of macrophages expressing trb-1 in the aortic roots in this model was 26.3 ± 1.19%, n = 5. The remaining medial cells positive for trb1 but which are not macrophages are most likely to be smooth muscle cells.

### 2.2. Trb2 Expression in Mouse Atherosclerotic Lesions

We have already shown that trb-2 is expressed in smooth muscle cells and endothelial cells cultured *in vitro* and that this changes little under conditions of inflammatory stimulation [17]. Recently, a role for trb-2 was proposed by Deng *et al.* in regulating cytokine expression in macrophages, resident in unstable plaques [15]. In line with this, we have shown that acetylated LDL potentiates LPS-induced IL-8 production in human monocytes [14]. Modified lipid downregulates trb-2 expression, leading to enhanced activation of ERK and JNK MAPK pathways. This, in turn, leads to a selective increase in IL-8 expression. 

**Figure 3:**
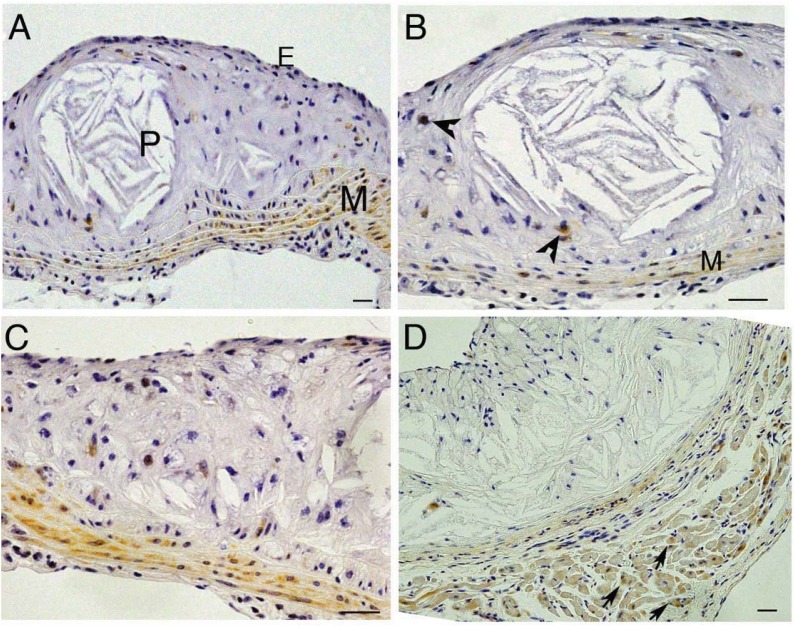
Tribbles 2 (trb2) expression in experimental mouse atherosclerosis. **A** Trb-2 expression in the aortic sinus, P, (plaque), M (media), E (endothelium); **B **high power of A, M (media), macrophages (arrows); **C** another high power image of a plaque (P) showing trb-2 expression in the media; **D** trb2 staining in the adventitial region of the aortic sinus to show positive staining in adipose tissue (arrows).

In the stable mouse atherosclerotic lesions investigated here, trb-2 appeared to be weakly expressed (Figure 3A, B) in smooth muscle cells and some macrophages (Figure 3C and D). Expression in adipose tissue in the adventitial area was also noted (Figure 3D).

### 2.3. Trb3 Expression in Mouse Atherosclerotic Lesions

**Figure 4:**
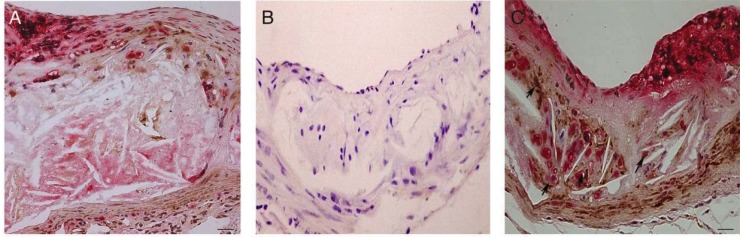
Tribbles 3 staining in experimental mouse atherosclerosis. **A **Dual staining of trb-3 (brown) and macrophages (Gal3); **B** negative control (secondary Ab only); **C** dual staining as A to show some coincident trb-3 and macrophage staining to show an equal proportion of trb3 in macrophages and VSMC.

*In vitro*, trb-3 is constitutively expressed in smooth muscle cells and endothelial cells and is induced up-to 6 fold after LPS stimulation [17]. Further, upregulation of trb-3 has been shown in hypoxia [18] and cellular stress [19]. However, the tissue distribution of this protein in the atherosclerotic vessel wall is currently unknown. In mouse atherosclerotic lesions, positive trb-3 staining was observed in smooth muscle cells (Figure 4A) and macrophages predominantly (Figure 4C).

### 2.4. Expression of Trbs in Mouse Atherosclerotic Lesions in the Absence of IL-1 Signalling

Since IL-1 is known to be critical in the development of atherosclerosis in mice [16,20] and IL-1 expression mirrors raised expression of trb-1 in vascular smooth muscle cells *in vitro* [13], we investigated whether the spatial expression of trbs were altered in atherosclerotic lesions from ApoE^−/−^IL1R1^−/−^ (DN) mice. We immunostained aortic root samples from DN mice after fat feeding for 8 weeks and determined the extent of trb expression in relevant cell types. There were no significant changes in expression of trb-2 and trb-3 in the absence of IL-1 signalling in the context of atherosclerosis (data not shown). 

**Figure 5:**
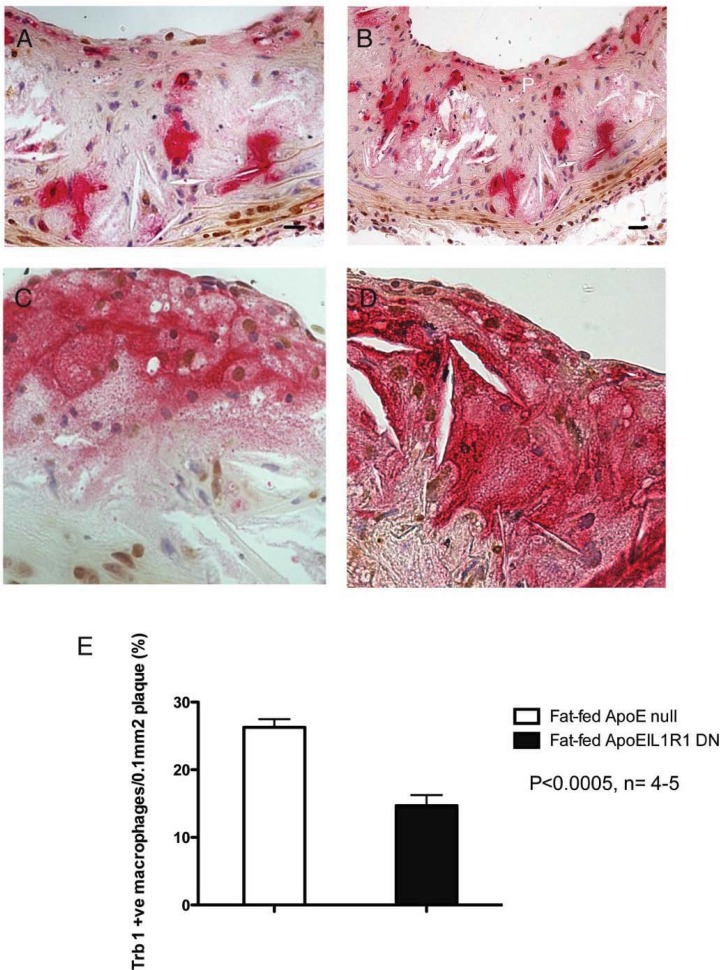
Macrophage tribbles 1 expression is reduced in experimental atherosclerosis in mice lacking the ability to signal via the IL-1 receptor. **A **and **B, **aortic plaques from ApoE^−/−^IL-1R1^−/−^ mice. Macrophages (red), trb-1 (brown). Note: the *non*-coincident staining for trb1 and macrophages in these sections in comparison to Figure 2; **C** and **D**, high power to show fewer macrophages positive for trb-1 in plaques than seen in plaques from ApoE^−/−^ mice (Figure 1 and Figure 2). **E** Quantitation of Trb 1 expressing macrophages per 0.1 mm2 of aortic root plaque in ApoE ^−/− ^*vs.* ApoE^−/−^IL-1R1^−/−^ mice.

However, in lesions from DN mice analysed for trb-1 positive staining, there were significantly less trb-1 positive macrophages/0.1mm^2^ of plaque than in ApoE^−/−^ animals (14.7% ± 1.55, n = 4 compared with 26.3% ± 1.19 [ApoE^−/−^], n = 5) (Figure 5E). Representative Figure 5A-C show that there are considerably less macrophages (red) that co-stain for trb-1 (brown) in aortic root plaques from ApoE^−/−^IL-1R1^−/−^ mice than from controls (Figure 5D) fed the same diet (Figure 2). The percentage of macrophages compared to the total number of cells within lesions was unchanged between the two strains 53 ± 4.1 ApoE^−/−^ vs. 42 ± 6 (ApoE^−/−^IL-1R1^−/−^) (n = 4 − 5, p = NS) as has been described previously [16]. In addition, we have not observed a substantial change in trb-1 expression in VSMC and endothelial cells between the two strains.

### 2.5. Tribbles-1 is a Negative Regulator of IL-6 Production and its’ Expression is Up-Regulated by IL-1 in Macrophages in vitro

**Figure 6:**
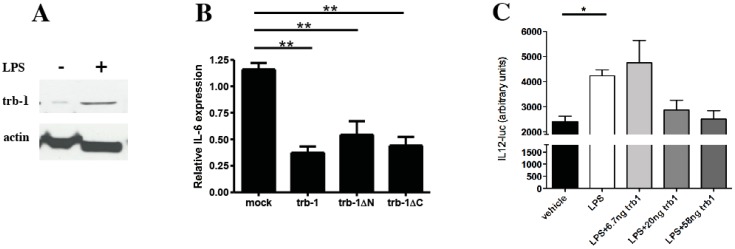
Trb-1 expression is induced by LPS and this protein controls IL-6 production in Raw 267.4 macrophages. **A.** Raw 264.7 cells were stimulated with 100 ng/mL LPS for 24 hrs and expression of trb-1 was monitored by western blot. As a loading control, expression of beta actin was determined, by stripping the membrane after probing for trb-1 and re-probing for the housekeeping control gene. A representative blot is shown from three independent experiments, with identical results. **B.** Raw 264.7 cells were stimulated 24 hrs post-transfection with 20 ng/mL LPS for 6 hrs and IL-6 expression measured in the media by ELISA. Three independent experiments were performed, with three biological replicates each time. As no IL-6 expression was observed in the absence of LPS treatment, only stimulated samples are shown. Expression levels were normalised to the mean value in the mock-transfected treatment for each experiments and the normalised data were pooled for statistical analysis. One-way ANOVA was performed to assess statistical significance of the results. ** p < 0.001. **C.** Raw 264.7 cells were transfected with an IL12-luciferase reporter in the presence or absence of overexpressed trb-1 and the activation of the reporter by LPS was monitored. One-way ANOVA was performed to assess statistical significance of the results. * p < 0.05.

In order to investigate the biological significance of tribbles-1 expression levels in macrophages (as seen in the ApoE^−/−^ aortic roots) and to determine if this was generally controlled by TIR receptor mediated inflammatory stimuli, first we established whether the Raw 264.7 macrophage cell line recapitulated our *in vivo* findings (Figure 2 and Figure 5). Raw 264.7 cells were stimulated by LPS for 24 hrs and the expression of trb-1 was determined by western blot. We chose LPS as an agonist in these experiments as Raw 264.7 cells are well known to be highly responsive to this proinflammatory compound. In addition, LPS signals through signalling pathways similar to those induced by IL-1. As the data in Figure 6A show, trb-1 expression is upregulated under these conditions.

Next, the functional consequences of overexpressed trb-1 were tested in Raw 264.7 cells, transfected with a trb-1 expression construct. Transfected cells were then stimulated with LPS and IL-6 production examined by ELISA. Our results demonstrate that overexpressed trb-1 strongly inhibits the production of IL-6 in macrophages (Figure 6B). Expression of truncated trb-1 mutants, lacking the proline rich N-terminal (aa 1-92) or the C-terminal (aa 338-372) region—as described before [21]—suggest that the kinase-like domain of this protein sufficient to exert inhibitory action. This is in line with our previously published mechanistic data on tribbles action [6,13]. To further elucidate the importance of trb-1 in the inflammatory activation of macrophages, Raw 264.7 cells were transfected with a luciferase reporter, driven by the promoter of the IL-12 gene, and the impact of an increasing dose of overexpressed trb-1 on LPS induced IL-12 expression was investigated. In line with the proposed role for trb-1 as an anti-inflammatory molecule, LPS induced IL-12 expression was abrogated in response to overexpressed trb-1.

## 3. Discussion

Molecular control of cellular function is achieved via the coordinated action of key intracellular signal processing systems. A number of these, including the MAPK signalling network and PI3K driven signal transduction pathways have been the subject of intense research in connection to cardiovascular disease and their potential as targets for therapeutic intervention [22,23].

The contribution of MAPK pathways has been elucidated recently. It has been shown that scavenger receptor (CD36) mediated signalling, triggered by oxLDL, is transmitted via src tyrosine kinases (lyn) and MAPK kinase kinase kinase (MAPKKK) proteins. Blocking lyn or JNK signalling via pharmacological inhibitors led to attenuated foam cell formation [24]. Atherosclerosis studies in gene deficient mice showed that JNK2 but not JNK1 deficiency led to reduced plaque size [25]. It has been suggested that this is due to the blockade of foam cell development via impaired scavenger receptor signalling. Similar to JNK, p38α has also been suggested to play a role in the control of CD36 mediated foam cell formation [24].

Cellular activation via the family of PI3K proteins has also been investigated in the context of atherosclerosis. Specifically, PI3Kγ and δ have been shown to control T cell migration, production of reactive oxygen species (ROS) in macrophages in response to proinflammatory stimuli, the secretion of proinflammatory cytokines, including IL-1 and IL-6 as well as activation of mast cells and platelet function [23]. 

The regulation of the MAPK and PI3K signalling pathways by tribbles family members is now well established [6,26]. However, very little is known about the expression profile of specific tribbles in atherosclerotic plaques, their contribution to disease development and the mechanisms that control tribbles expression in this setting. 

To address these issues, we performed a systematic analysis of the expression pattern of all three mammalian tribbles in the ApoE^−/−^ fat-fed model of atherosclerosis and show that these proteins are present in smooth muscle cells, endothelial cells and plaque resident macrophages. 

IL-1 is widely appreciated as a major contributor of plaque development in atherosclerosis. In line with this, ApoE/IL-1R double knockout mice are protected from atherosclerosis, compared to ApoE^−/−^ controls [16]. Given previous findings that tribbles-1 expression correlates with induction of IL-1 expression in vascular cells and that the expression of this gene family is regulated by inflammatory stimuli in a cell-type specific manner, we hypothesised that elimination of the IL-1 driven inflammatory stimulus (by knocking out the IL-1 receptor) would modulate tribbles expression in atherosclerotic plaques. This is shown in this study selectively for macrophage specific expression of tribbles-1. Based on these data and the above literature, we propose that tribbles-1 is expressed in plaque resident macrophages as part of an IL-1 induced negative feedback mechanism to control the extent of inflammatory activation of these cells. Our *in vitro* data support this model and suggests a negative regulatory role for this protein in the expression of proinflammatory cytokines in macrophages. This concurs with phenotypic analysis of macrophages from trb-1 deficient mice, which display an enhanced proinflammatory response to LPS [27]. We surmise therefore that impaired tribbles-1 expression *in vivo* due to genetic variations in the human population may sensitise macrophages to inflammatory stimuli and that this could contribute to the development of chronic inflammatory disease.

Our observations may provide a mechanistic explanation for the rapidly increasing genetic evidence that trb-1 is a significant risk factor in cardiovascular disease [28,29]. Whilst the molecular mechanisms for such a role are still not well understood, two lines of supporting evidence have been put forward. First, genome wide association studies (GWAS) identified trb-1 as a risk factor in hyperlipidaemia and MI [28] and, second, Ostertag revealed that trb-1 is a critical controller of inflammatory responses in adipocytes [30]. In both settings, reduced tribbles-1 accompanied elevated inflammatory responses, agreeing with our proposed model of trb-1 mediated control of cellular homeostasis.

Tribbles-2 and -3 have also been detected in specific cells of the plaque. A recent study published by Deng *et al* [15] suggested that trb-2 expression in plaque resident macrophages correlated with plaque vulnerability and this may be of functional importance in the onset of ACS. Whilst our experiments were not designed to address this question (the majority of plaques were stable), we did detect the expression of trb-2 in macrophage-like cells in the plaque core. This is in agreement with data showing that oxLDL induced upregulation of trb-3 expression in monocyte-derived macrophages [31]. 

In addition to macrophages and smooth muscle cells, recent reports have suggested a role for tribbles in the control of adipocyte function [26,30,32]. We routinely detected expression of tribbles in adipocyte-like cells in the far adventitial area of vessels lending some observational support to these previous findings. 

## 4. Experimental Section

### 4.1. Animals

ApoE^−/−^ mice (stock no. 002052) and IL-1R1^−/−^ (stock no. 3245) were obtained from the Jackson Laboratories; ApoE^−/−^ IL-1R1^−/−^ double deficient mice were generated by cross-breeding. All mice were on a C57BL/6 background and fed normal ‘chow’ (4.3% w/v fat, 0.02% w/v cholesterol) or ‘Western’ (18.5% w/v fat, 0.9% w/v cholesterol, 0.5% w/v cholate) diet. Animals were housed in a controlled environment with a 12 hour light/dark cycle at 22 °C. All experiments were performed in accordance with UK legislation under the 1986 Animals (Scientific Procedures) Act, and approval was granted by the University ethics review board. The investigation conforms with the Guide for the Care and Use of Laboratory Animals published by the US National Institutes of Health (NIH Publication No. 85-23, revised 1996). 

### 4.2. Cell Culture

Raw 264.7 cells (ATCC: TIB-71) were cultured in standard media and treated with of LPS, as specified in the appropriate figure legends. 

### 4.3. Western Blot, ELISA

Trb-1 protein was detected using a trb-1 antibody (Millipore) diluted 1:500 in 0.05% v/v Tween 20/5% v/v non-fat milk as previously described [13]. Briefly, membranes were blocked in 0.05% v/v Tween 20/5% v/v non-fat milk for 2 hours at RT, then incubated overnight at 4ºC with a polyclonal trb-1 specific antibody (Millipore 09-126) diluted 1:500 in blocking buffer, and detected by a HRP-conjugated goat anti-rabbit IgG secondary antibody (Dako, P0448). Membranes were developed with SuperSignal West Pico chemiluminescent substrate, and signals were quantified by Chemigenius gel documentation system (Syngene).

ELISA was performed to quantify the production of mouse IL-6 (R&D systems), as recommended by the manufacturer.

### 4.4. Immunohistochemistry

Paraffin-embedded sections of aortic sinus were stained immunohistochemically for smooth muscle actin (Dako, M0851, 1:150) to visualize vascular smooth muscle cells and Galectin-3 (R&D, AF1197, 1:50) to visualize macrophages. Trb antibodies used were: trb-1 (Millipore, 09-126) at 1:125, trb-2 (Sigma, HPA001305) at 1:200 and trb-3 (Millipore) at 1:200.

Standard immunohistochemical techniques were applied.

### 4.5. Analysis of Trb Expression in Atherosclerotic Lesions

The number of different types of cells expressing trbs was assessed by morphometric measurement of histologically stained paraffin-embedded sections using a Lucia image analysis software system (Lucia G, Nikon, UK). The total and positively stained cell nuclei were counted within a fixed area. The values reported represent a mean from at least 5 sections for each animal. 

## 5. Conclusions

In summary, this is the first systematic study of tribbles expression in experimental atheroma and the data suggest an important regulatory function for tribbles-1 in vascular cell pathophysiology. In addition, our data clearly demonstrate that inflammatory signals selectively control the expression of trb-1 in plaque macrophages, suggesting that this may be an important homeostatic control mechanism to modulate the inflammatory phenotype of these cells.
